# Evaluation of the clinical course of immune-mediated polyneuropathy in cats after treatment with human intravenous immunoglobulins

**DOI:** 10.1177/1098612X261478277

**Published:** 2026-08-03

**Authors:** Annika Katharina Schmitz, Alexander Paul Sablotni Aguilera, Gesine Buhmann, Jana van Renen, Birgit Parzefall, Kaspar Matiasek, Lara Alexa Matiasek, Andrea Fischer

**Affiliations:** 1 Centre for Clinical Veterinary Medicine, Neurology Unit, Ludwig-Maximilians-Universität München, Munich, Germany; 2 Neurology Department, Small Animal Clinic Oberhaching, Oberhaching, Germany; 3 AniCura Tierklinik Haar GmbH, Neurology Department, Haar, Germany; 4 Section of Clinical and Comparative Neuropathology, Centre for Clinical Veterinary Medicine, Ludwig-Maximilians-Universität München, Munich, Germany

**Keywords:** weakness, immunotherapy, lower motor neuron disease, tetraparesis, non-ambulatory

## Abstract

**Objectives:**

Immune-mediated polyneuropathy (IMPN) manifests with generalised lower motor neuron (LMN) weakness and a remittent or relapsing clinical course. Evidence-based treatment recommendations for severely or chronically affected cats are limited. The objective of this study was to evaluate the clinical course and outcome of cats with IMPN after treatment with human intravenous immunoglobulins (hIVIg).

**Methods:**

Medical records from two veterinary referral hospitals were reviewed (2018–2025) for cats with IMPN treated with hIVIg. Cats were treated with 1–2 g/kg hIVIg divided over 2–4 days (0.5 g/kg/day). Clinical information, diagnostic findings, treatment details, and short- and long-term follow-up were evaluated retrospectively. Follow-up information was obtained from medical records and owner communication. Minimum follow-up duration was 8 months (range 8–51; median 17). Clinical course (partial and complete recovery, relapses) was compared between cats with acute/recent onset (four cats) or chronic presentation (five cats).

**Results:**

Of nine cats, eight exhibited clinical improvement after hIVIg administration, achieving ambulatory status (walking >5 steps) within a median of 4 days (range 2–8). One cat failed to respond to hIVIg but improved after subsequent prednisolone therapy. For the overall cohort, the median time to complete recovery was 20 days, while the median time to partial recovery was 14 days. Relapses occurred in four cats, but weakness was less severe, and all cats recovered again. The remaining five cats remained relapse-free.

**Conclusions and relevance:**

Human IVIg was well tolerated in this small cohort, and rapid clinical improvement was observed in most treated cats. These findings suggest that hIVIg may be considered as a treatment option in selected cats with IMPN, including those with acute and chronic presentations. Larger prospective studies, including comparison with no treatment or corticosteroids, are warranted to further assess its efficacy.

## Introduction

Immune-mediated polyneuropathy (IMPN) is increasingly reported worldwide in young cats.^1–3^ The leading clinical sign is lower motor neuron (LMN) weakness. Affected cats develop paraparesis, tetraparesis or tetraplegia.^1–3^ The diagnosis is usually made based on clinical signs, history and clinical course, ruling out other causes of generalised weakness, and in some cases, supportive diagnostic tests targeting peripheral nerves (electrodiagnostics, biopsies). Not all cats will have all these tests. This is similar to the analogous conditions in humans and dogs.

Half of all affected cats show a remittent clinical course; others experience relapses.^1^ The clinical signs and course, electrodiagnostic studies, anti-GM2 and anti-GalNAc-GD1a IgG antibodies, and nerve biopsies show parallels to IMPNs in humans, such as Guillain–Barrè syndrome (GBS)^4,5^ and chronic inflammatory demyelinating polyneuropathy (CIDP),^6–10^ and acute canine polyneuropathy (ACP)/polyradiculoneuritis in dogs.^3,11–14^

Human intravenous immunoglobulin (hIVIg) is a 10% liquid preparation containing ⩾99% human IgG and is licensed for the treatment of various immune-mediated disorders in humans.^15^ The current guidelines of the European Academy of Neurology/Peripheral Nerve Society recommend hIVIg as a treatment for CIDP^16^ and GBS.^17^ Human IVIg is not approved for veterinary use but has been successfully administered off-label for immune-mediated diseases in dogs and less commonly in cats.^13,18,19^ Human IVIg is also mentioned as a potential treatment in the recent American College of Veterinary Internal Medicine consensus statement on immune-mediated thrombocytopenia.^20^ Reports describe hIVIg treatment in cats with immune-mediated haemolytic anaemia (IMHA) and in a case of severe erythema multiforme.^19,21,22^

Treatments for feline IMPN, such as L-carnitine supplementation and corticosteroids, have been explored but have failed to show a consistent beneficial effect.^1,2^ Evidence-based treatment recommendations for cats with severe or chronic manifestations of IMPN are currently lacking.^1,2^

In this cohort, hIVIg was administered as a treatment in cats with IMPN with severe neurological impairment or chronic manifestation. The aim of this article was to describe the short- and long-term outcomes.

## Materials and methods

### Case recruitment

Medical records from two veterinary hospitals were searched (2018–2025) for cats who underwent treatment with hIVIg. In total, 20 cats who had received hIVIg therapy were identified: four cats had IMHA and 16 cats had immune-mediated neurologic disease (IMPN 12, polymyositis 1, myasthenia gravis 1, suspected dysautonomia 1, limbic encephalitis 1). Two cats were excluded because IMPN was not regarded as the primary suspected cause of their LMN weakness. In addition, one cat with suspected IMPN was excluded owing to a lack of follow-up information. Nine cats with IMPN who received hIVIg and had long-term follow-up were included. All cats were evaluated by a veterinary neurologist (European College of Veterinary Neurology diplomate or resident).

### Outcome assessment

Medical records were reviewed for results of history, neurological and diagnostic examinations, severity of paresis at each examination, outcome and relapses. Cat owners were also contacted by telephone or email to assess long-term follow-up. Median follow-up duration was 17 months (range 8–51). A neurological grading system (Table 1) was created to retrospectively grade the severity of paresis at each examination or follow-up report. Partial recovery was defined as the achievement of grade 6 and complete recovery as the achievement of grade 7. Time span (weeks) from presentation to recovery (median) and relapse rates were assessed for all cats and cats with acute or chronic clinical signs and compared with retrospective data from two previously published cohorts of cats with IMPN who did not receive this treatment.^1,2^ Comparison with previous studies was descriptive. Owing to the small sample size of the present cohort, the retrospective design, lack of individual patient-level data from previous studies and differences in outcome definitions, formal statistical comparison with published cohorts was not performed.

**Table 1.** Clinical grading system used to assess weakness and functional recovery in cats with immune-mediated polyneuropathy.

Grade	Clinical description
0	Requiring ventilatory support
1	Tetraplegia
2	Paraparesis or tetraparesis; non-ambulatory
3	Paraparesis or tetraparesis; briefly ambulatory (<5 steps)
4	Ambulatory (>5 steps)
5	Ambulatory (>20 steps)
6	Playing >30 mins/day (partial recovery)
7	Jumping (complete recovery)

## Results

### Animals

The study cohort comprised nine cats. Information on signalment, treatments administered before presentation, clinical course, neurological grade and diagnostic work-up are summarised in Table 2. Seven cats were male (two intact, five neutered) and two cats were female neutered. The median age at presentation was 9.5 months (range 6–32) in eight cats. One cat (British Shorthair, male neutered) was 11 years old. The distribution of breeds was as follows: Maine Coon (3/9), Bengal (2/9), British Shorthair (2/9), Birman-mix (1/9) and domestic shorthair (1/9).

**Table 2.** Signalment and diagnostic work-up in nine cats with immune-mediated polyneuropathy.

	Acute/recent onset				Chronic presentation				
	Cat 1	Cat 2	Cat 3	Cat 4	Cat 5	Cat 6	Cat 7	Cat 8	Cat 9
Signalment	Age 32 months; FN; BSH	Age 7 months; MN; Birman-mix	Age 6 months; MN; Maine Coon	Age 9 months; MN; DSH	Age 9 months; MI; Maine Coon	Age 10 months; MN; Maine Coon	Age 20 months; FN; Bengal	Age 11 months; MI; Bengal	Age 11 years; MN; BSH
Clinical course before hIVIg	3 days	3 days	4 days	7 days	1 month	5 months	3 months	3.5 months	12 months
Treatment/supplements before presentation	–	B vitamins, L-carnitine, coenzyme Q	–	–	–	NSAID (meloxicam)	B vitamins, L-carnitine	B vitamins, L-carnitine	NSAID (meloxicam); B vitamins, L-carnitine coenzyme Q
Neurological grade at presentation	1	2	2	3	2	2	3	3	3
Laboratory results	CBC: unremarkable; blood gas: lactate 3.3 mmol/l; serum chemistry: unremarkable; CK: 415,9 U/l; Toxoplasma Ab IgG: low; IgM: borderline; FIV/FeLV: negative; AChR-Ab: negative	CBC: unremarkable; blood gas: iCa: 1.5 mmol/l; serum chemistry: unremarkable; CK: 488 U/l; Toxoplasma Ab IgG/IgM: negative; FIV/FeLV: negative	CBC: unremarkable; blood gas: iCa: 1.5 mmol/l; serum chemistry: unremarkable; CK: 488 U/l; FIV/FeLV: negative; Toxoplasma Ab IgG/IgM: negative	CBC: unremarkable; serum chemistry: unremarkable; CK: unremarkable; Toxoplasma Ab IgG/IgM: negative; FIV/FeLV: negative	CBC: mild leucocytosis; serum chemistry: unremarkable; CK: unremarkable; Toxoplasma Ab IgG/IgM: negative	CBC: unremarkable; serum chemistry: unremarkable; CK: unremarkable; AChR-Ab: negative	CBC: unremarkable; blood gas: iCa:1.4 mmol/l unremarkable; serum chemistry: unremarkable	CBC: mild leucocytosis; CK: unremarkable	CBC: unremarkable; serum chemistry: unremarkable; CK: 557 U/l
CSF	✓	–	–	✓	–	–	–	–	–
MRI	–	–	✓	✓	–	–	–	–	✓
Electromyography examination	✓	✓	✓	✓	–	–	–	–	✓
Muscle nerve biopsies	✓	–	–	✓	–	–	–	–	✓

*Note*. ✓: performed/administered; –: not performed/not administered; Ab: antibody; AChR-Ab: acetylcholine receptor antibodies; BSH: British Shorthair; CBC: complete blood count; CK: creatine kinase; CSF: cerebrospinal fluid; DSH: domestic shorthair; FeLV: feline leukaemia virus; FIV: feline immunodeficiency virus; FN: female neutered; hIVIg: human intravenous immunoglobulin; iCa: ionised calcium (reference interval 1.25–1.35 mmol/l); MI: male intact; MN: male neutered; NSAID: non-steroid anti-inflammatory drug.

### History and clinical presentation

All cats presented with generalised or pelvic limb weakness and were either non-ambulatory or only briefly ambulatory. Four cats (cats 1–4) presented with acute/recent onset and severe clinical signs (⩽1 week) and five cats (cats 5–9) presented with chronic clinical signs (⩾1 month). None of the cats had been treated with corticosteroids. General physical examination findings were unremarkable in all cats.

In the acutely affected cats, the first signs of weakness occurred at a median of 3.5 days (range 3–7) before presentation.

In the chronically affected cats, the first signs were recognised a median of 3.5 months before presentation (1 month, 3 months, 3.5 months, 5 months and 12 months). Owners of the chronically affected cats reported the initial clinical signs as weakness, particularly affecting the pelvic limbs, characterised by reluctance to jump, plantigrade stance, short-stepped gait and the need for frequent rest while walking. These cats showed gradual progression of paresis over more than 1 month, with subtle fluctuations. Owners indicated that the quality of life was substantially impaired in all cats. In addition, two chronically affected cats had been previously treated with non-steroidal anti-inflammatory drugs, without improvement of clinical signs. Four cats had received nutritional supplements (e.g. L-carnitine or B vitamins).

### Neurological examination

The neuroanatomic lesion localisation was generalised LMN in all cats (Figure 1; Videos 1 and 2 in the supplemental material). At presentation, five cats (cats 1–6) were non-ambulatory and four cats (cats 4, 7–9) were only briefly ambulatory. The briefly ambulatory cats were able to walk only two or three steps, showed a stiff or stilted gait, and then frequently sat or lay down.

**Figure 1.**
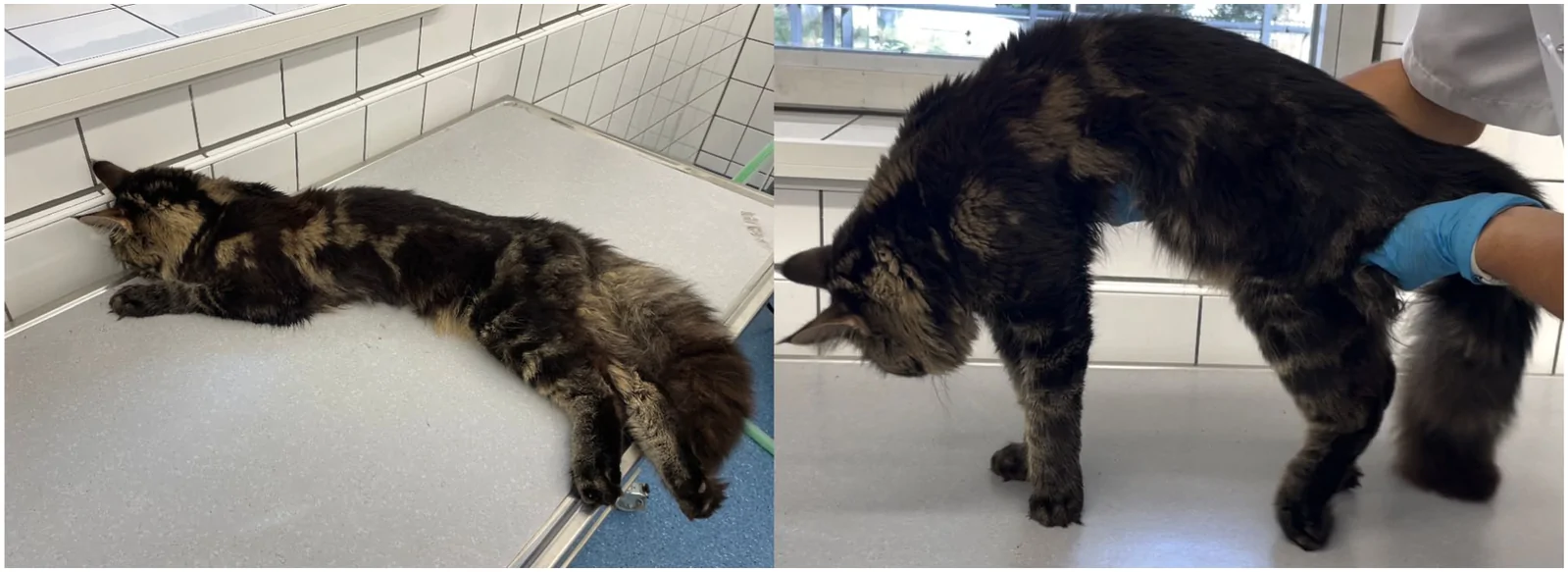
Clinical presentation of a chronically affected cat (cat 6) with immune-mediated polyneuropathy showing non-ambulatory lower motor neuron tetraparesis (grade 2) at presentation (day 0).

Of the acutely affected cats, two were non-ambulatory tetraparetic, one was tetraplegic and one was only briefly ambulatory with loss of independent ability to urinate and defecate.

Overall, neurological examination findings showed generalised LMN dysfunction in all nine cats, with paraparesis in one cat, tetraparesis in seven cats and tetraplegia in one cat; withdrawal reflexes were decreased or absent in all affected limbs. Other findings were plantigrade stance (4/9), flexion of the head and neck (3/9), ataxia (2/9), decreased proprioceptive paw positioning and hopping in pelvic limbs (6/9) and thoracic limbs (4/9), decreased patellar reflexes (4/9) and spinal hyperesthesia on palpation (5/9). All abnormalities were symmetrical. Mental status and cranial nerve examination were unremarkable in all cats, and none had a change in meow or dysphagia. The 11-year-old cat (cat 9) who presented with a chronic course over 12 months also exhibited pronounced atrophy of the pelvic limb muscles.

### Diagnostic work-up

Detailed information on diagnostic investigations is provided in Tables 2 and 3.

**Table 3.** Advanced diagnostic findings in five cats with immune-mediated polyneuropathy.

	Acute/recent onset				Chronic presentation
	Cat 1	Cat 2	Cat 3	Cat 4	Cat 9
MRI	Not performed	Not performed	1.5 T (Magnetom Symphony; Siemens); sequences included sagittal T2W, dorsal plane STIR, dorsal plane T2W, and transverse T2W and transverse T1W images before and after contrast administration; coverage: T3–sacrum; FoV 25 × 25 cm. Findings: no abnormalities	1.5 T (Magnetom Symphony; Siemens); sequences included sagittal T2W, dorsal plane STIR, dorsal plane T2W, and transverse T2W and T1W images before and after contrast administration; coverage: C4–sacrum; FoV 44 × 44 cm. Findings: no abnormalities	0.4 T (Aperto; Hitachi); coverage: C1–sacrum; FoV 25 × 25 cm. Findings: mild intervertebral disc protrusion at C5/6 and T13/L1, with mild reduction of the dorsal CSF signal and no intramedullary signal change
CSF	TCC: 2/ul; protein concentration: 0.05 g/l; cytology: unremarkable	Not performed	Not performed	TCC: 2/ul; protein concentration: 0.13 g/l; cytology: unremarkable	Not performed
Electrodiagnostic findings	EMG: fibrillation potentials and positive sharp waves in thoracic and pelvic limb muscles MNCV: not assessable owing to increased stimulation threshold CMAP: decreased amplitude. Repetitive nerve stimulation at 3 Hz: decrement from first to fifth response >10%	EMG: fibrillation potentials and positive sharp waves in proximal thoracic and pelvic limb muscles and tongue MNCV: tibial nerve 76 m/s; ulnar nerve 64 m/sCMAP: decreased amplitude with temporal dispersion. Repetitive nerve stimulation: no decrement	EMG: fibrillation potentials and positive sharp waves in thoracic and pelvic limb muscles and tongue MNCV: tibial nerve 76 m/s; ulnar nerve 64 m/s CMAP: decreased amplitude with temporal dispersion. Repetitive nerve stimulation: no decrement	EMG: spontaneous electrical activity in multiple muscles, more pronounced in pelvic than thoracic limbs MNCV: ulnar nerve >70 m/s; tibial nerve >70 m/s CMAP: decreased amplitude with temporal dispersion. Repetitive nerve stimulation at 3 Hz: no decrement	EMG: fibrillation potentials and positive sharp waves in most pelvic and thoracic limb muscles, paraspinal muscles and tongue; occasional complex repetitive discharges MNCV: tibial nerve 89.7 m/s; ulnar nerve 88.2 m/s CMAP: decreased amplitude with temporal dispersion F-waves: tibial nerve absent. Repetitive nerve stimulation at 3 Hz: no decrement
Biopsied nerve/muscle	Biceps femoris muscle, cranial tibial muscle and fibular nerve	Not performed	Not performed	Biceps femoris muscle, cranial tibial muscle and fibular nerve	Cranial tibial muscle, saphenous nerve and radial nerve
Histopathological findings	Muscle: mixed-type muscle atrophy with denervation Nerve: severe multifocal lymphocytic terminal branch neuritis and mild subacute lymphocytic inflammatory neuropathy	Not applicable	Not applicable	Muscle: diffuse, chronic, moderate-to-severe denervation-type atrophy, partially compensated Nerve: marked lymphohistiocytic, predominantly paranodal, large fibre-dominant inflammatory neuropathy	Muscle: severe diffuse chronic denervation-type atrophy, partially compensated, and moderate chronic fibre-depleting intramuscular neuropathy Nerve: mild chronic oligofocal demyelinating neuropathy, more pronounced in the saphenous than radial nerve

Reference intervals for feline motor nerve conduction velocity (MNCV) were taken from Mizisin et al.^23^

*Note*. CMAP: compound muscle action potential; CSF: cerebrospinal fluid; EMG: electromyography; FoV: field of view; STIR: short tau inversion recovery; T1W: T1-weighted; T2W: T2-weighted; TCC: total cell count.

Haematology and serum chemistry were performed in all cats. No relevant findings were identified. Two cats showed a borderline increase in ionised calcium.

The serum activity of creatine kinase was available in 8/9 cats and was mildly elevated in four cats. Acetylcholine receptor antibodies were tested in three cats and were negative (reference interval <0.3 nmol/l; Neuromuscular Diagnostic Laboratory, University of California, San Diego, USA). *Toxoplasma gondii* antibodies IgG and IgM were tested in five cats, and feline immunodeficiency virus (FIV) and feline leukaemia virus (FeLV) in four cats. *T gondii* testing revealed negative IgG and IgM titres in four cats, while one cat had a low IgG titre and a borderline IgM titre. FIV antibody and FeLV antigen tests were negative in all tested cats (4/4).

Five cats underwent general anaesthesia and diagnostic testing. Electrodiagnostic studies were carried out in five cats (cats 1, 2, 3, 4 and 9) and muscle/nerve biopsies in three cats (cats 1, 4 and 9). Electromyography identified generalised spontaneous electric activity characterised as positive sharp waves and fibrillation potentials in all five cats. Complex repetitive discharges were additionally detected in one cat (Table 3). Electromyographic abnormalities in cat 1 were suggestive of either a more prolonged clinical course than the 3-day history reported by the owner or a concurrent myopathy. In all cats, tibial nerve assessment revealed temporal dispersion, and decreased amplitude of the evoked compound muscle action potential and decreased motor nerve conduction velocity in one cat. The results of the electrodiagnostic examination were consistent with a distal demyelinating polyneuropathy or a mixed demyelinating and axonal polyneuropathy.

Muscle and nerve biopsies were performed in three of these cats (Table 3). Findings were indicative of IMPN.^24^ The nerve biopsies revealed sequentially widened nodes of Ranvier and dysmorphic paranodal regions with lymphohistiocytic infiltration and demyelination. In addition, fibre-adherent and partially invasive inflammatory cells at the Schmidt–Lanterman clefts were found in two cats. In the 11-year-old cat (cat 9), histopathological findings were consistent with a late or post-inflammatory immune-mediated polyradiculoneuropathy, with major trunks mildly affected and active infiltrates possibly limited to terminal intramuscular branches. Muscle biopsies revealed diffuse chronic denervation-type atrophy with myofibre heterogeneity, atrophy clusters and endomysial fibrosis. MRI of the vertebral column and spinal cord was performed in three cats (cats 3, 4 and 9). Cerebrospinal fluid (CSF) analysis was performed in two cats (cats 1 and 4). No clinically significant abnormalities were identified on MRI or CSF analysis (Table 3).

### Treatment with hIVIg

Figure 2 illustrates the clinical course after hIVIg treatment. Cats were treated with hIVIg at a dosage of 1–2 g/kg IV divided over 2–4 days (0.5 g/kg/day). The treatment regimen and clinical course after hIVIg for each cat are summarised in Table 4. Each cat was hospitalised and treated with hIVIg (Privigen; CSL Behring) as a continuous rate infusion (CRI) for 6 h each day, while the cats were constantly monitored. A new vial was opened each day, and the infusion was carried out under light-protected conditions using a light-shielding vial holder and light-protected infusion tubing. A lower infusion rate was applied at the start of the CRI. The cats’ vital signs were monitored every 5 mins during the first hour and every 15–30 mins for the following 5 h. Monitoring included body core temperature, mucous membranes, heart rate, respiratory rate and femoral pulses documented on dedicated documentation sheets for transfusions. Three cats received the hIVIg CRI on 2 consecutive days (cats 4, 7 and 8), five cats on 3 consecutive days (cats 2, 3, 5, 6 and 9) and one severely affected tetraplegic cat on 4 consecutive days (cat 1). Neurological examinations were performed q12h during hospitalisation, and the neurological examination was documented in the medical records each day. The cats were discharged after completion of the hIVIg protocol. Neurological status was documented during follow-up visits at the veterinary hospital (cats 1, 2, 3, 6, 7 and 9) or by telephone/email contact (cats 4, 5 and 8). All cats received B vitamins, L-carnitine and coenzyme Q from the time of presentation as part of a standard supportive treatment protocol for cats with LMN disease. In total, three cats received prednisolone (1 mg/kg, with subsequent tapering): cats 4 and 6 because of a relapse and cat 9 (non-responder) for lack of response to hIVIg. At the time of manuscript preparation, cats 6 and 9 continued to receive a low maintenance dose of prednisolone (0.2 mg/kg every third day).

**Figure 2.**
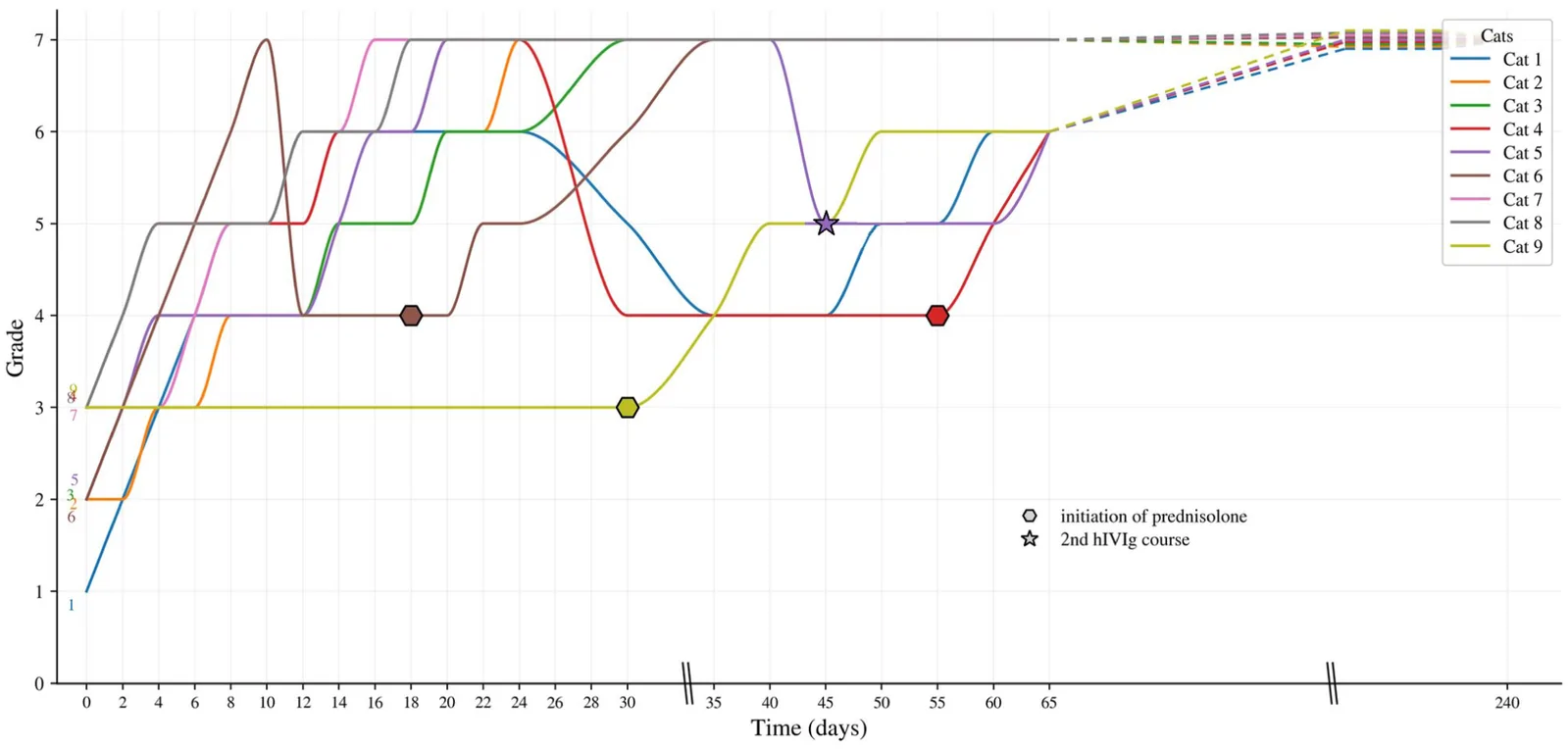
Clinical course and neurological grade after human intravenous immunoglobulin (hIVIg) treatment in nine cats with immune-mediated polyneuropathy.

**Table 4.** Human intravenous immunoglobulin (hIVIg) treatment and clinical outcome in nine cats with immune-mediated polyneuropathy.

	Acute/recent onset				Chronic presentation				
	Cat 1	Cat 2	Cat 3	Cat 4	Cat 5	Cat 6	Cat 7	Cat 8	Cat 9^a^
Daily hIVIg dose (g/kg)	0.5	0.5	0.5	0.5	0.5	0.5	0.5	0.5	0.5
No. of hIVIg treatment days	4	3	3	2	3	3	2	2	3
Total hIVIg dose (g)	10	6.3	7.5	5	7.5	7.5	5	5	7.5
Supplements started concomitantly with hIVIg	B vitamins (100 mg PO q24h), L-carnitine (500 mg PO q24h) and coenzyme Q (100 mg PO q24h) to all cats								
Grade at hIVIg initiation	1	2	2	3	2	2	3	3	3
Grade on last hIVIg day	3	3	4	4	4	4	3	4	3
Days until grade 4	6	8	4	2	4	4	6	2	35
Days until grade 6	14	20	20	14	16	8	12	12	50
Days until grade 7	>65^b^	24	30	20	20	9	16	18	>65^b^
Relapse after hIVIg	26 days	No	No	28 days	43 days	10 days	No	No	No
Treatment administered for relapse	None			Prednisolone (1 mg/kg PO q24h), started 55 days after hIVIg	2nd hIVIg course, started 50 days after 1st hIVIg	Prednisolone (1 mg/kg PO q24h), started 18 days after hIVIg	–	–	Prednisolone (1 mg/kg q24h), started 30 days after hIVIg
Follow-up (months)	18	12	12	18	51	27	8	17	11

a Cat 9 was 11 years old at presentation.

b >65 days: cat recovered completely (grade 7), but the exact time point was not documented.

### Short-term outcome

The severity of neurological impairment varied among the cats at the initiation of treatment with hIVIg on day 0, reflecting variable starting grades. One cat presented with LMN tetraplegia (grade 1), four cats with non-ambulatory LMN tetraparesis (grade 2), three cats with LMN tetraparesis and one cat with LMN paraparesis who was able to walk two to four steps (grade 3). All cats tolerated hIVIg CRI well, and no side effects were observed. Out of nine cats, eight showed improvement in weakness after treatment with hIVIg (responder); one cat showed no improvement (cat 9, non-responder). The responder cats improved for at least one grade within 1–6 days (median 2) after the treatment with hIVIg was initiated: these cats achieved grade 4 and were ambulatory and able to walk more than five steps without support within 2–8 days (median 4; acutely affected cats 5 days, chronically affected cats 4 days); the median time to grade 5, being able to walk for more than 20 consecutive steps without support, was 9 days (range 4–14). In the responder cats, the median time to partial recovery (grade 6), being able to play actively for more than 30 mins per day, was 14 days (range 12–20). The median time to complete recovery (grade 7), being able to jump, was reported after a median of 19 days (range 16–30).

The cat (cat 9) who failed to improve after hIVIg (non-responder) was subsequently treated with prednisolone (1 mg/kg, subsequently tapered to 0.5 mg/kg q48h) and improved to grade 6 approximately 20 days after initiation of prednisolone.

Video 3 (in the supplemental material) shows cat 6 at 4 days after initiation of hIVIg.

### Relapses

Relapses occurred in 4/8 responder cats (two acutely, two chronically affected) (Figure 2; Table 4). Weakness recurred 10 days (cat 6), 26 days (cat 1), 28 days (cat 4) and 43 days (cat 5) after completion of the hIVIg treatment course. The owners reported similar signs as described in the initial episode, although generally milder. All cats remained ambulatory (cats 1, 4 and 6: grade 4; cat 5: grade 5). No standardised retreatment protocol was applied. Cat 5 received a repeated course of hIVIg 46 days after the initial protocol. It took approximately 3 weeks until the cat reached grade 6 (partial recovery) again, compared with 16 days after the first hIVIg course. No side effects were observed. Cat 1 presented to the neurology service when the relapse occurred and underwent a neurological examination, which confirmed generalised LMN dysfunction. No further treatment was given and the cat recovered spontaneously after 34 days (grade 6, partial recovery).

Cats 4 and 6 were treated with prednisolone after relapse (55 and 18 days after hIVIg administration) and recovered again after 15 and 12 days, respectively (grade 6 partial recovery). The cats recovered completely (grade 7) on long-term follow-up.

### Long-term outcome

All cats (cats 1–9) recovered from IMPN. The median time from presentation to complete recovery was 20 days in the overall cohort: 18 days in chronically affected cats and 27 days in cats with an acute/recent onset (Table 5). The median follow-up duration was 17 months (range 8–51). Mild residual deficits persisted in two cats but were not apparent to the owner. Long-term follow-up data were available from on-site examinations or telephone/email contact. Cats who were examined on site had mild residual deficits such as a subtle decrease in withdrawal reflexes. In addition, two cats were able to jump, but not as high as before, which was considered insignificant by the cat owners.

**Table 5.** Comparison of recovery times with previously published cohorts.

Cohort/study	n	History	Ambulatory (>5 steps)	Partial recovery playing >30 mins	Complete recovery jumping	Recovery	Relapse rate
All cats	9	1 mo (3 days–12 mo)	4 days (2–35)	14 days (8–50)	20 days (9– >65)^a^	89% hIVIg; 11% prednisolone	44% (4/9)
Acute/recent onset	4	3.5 days (3–7)	5 days (2–8)	17 days (14–20)	27 days (20–>65)^a^	100% hIVIg	50% (2/4)
Chronic	5	3.5 mo (1–12)	4 days (2–35)^b^	12 days (8–50)^b^	18 days (9–>65)^ab^	80% hIVIg; 20% prednisolone	40% (2/5)
Van Caenegem et al. (2023)^2^	43	2 weeks (IQR 1–8)	–	–	4 weeks (IQR 3–10)	86% complete; 12% partial	23%
van Renen et al. (2022)^1^	63	1 mo (1–180)	–	–	1.5 mo (few days–17 mo)	79% complete or partial	36%

Data are median (range) unless otherwise indicated. For the present cohort, partial and complete recovery correspond to grades 6 and 7, respectively.

a >65 days: cats recovered completely (grade 7), but the exact time point was not documented.

b One 11-year-old cat was a non-responder who required 35 days to reach grade 4 and recovered subsequently with prednisolone treatment (grade 6, 50 days; grade 7, >65 days).

*Note*. hIVIg: human intravenous immunoglobulin; IQR: interquartile range; mo: months.

Cat 3 was admitted to the hospital on emergency 1 year later and died acutely from suspected feline infectious peritonitis (FIP) with Rivalta-positive abdominal effusion. Neurological examination had been normal 2 weeks after hIVIg treatment in this cat. According to the owner, the cat remained neurologically normal until presentation for the subsequent illness suspected to be FIP.

### Comparison with retrospective controls

Recovery times and relapse rates of all cats are summarised in Table 5 and compared with those in previously reported cohorts.

The median time from presentation to partial recovery was 14 days, and to complete recovery 20 days in the present cohort. Previous studies reported slightly longer median recovery times of 4 and 6 weeks, respectively.^1,2^ Notably, chronically affected cats who were treated with hIVIg achieved partial and complete recovery after a median of 12 and 18 days, respectively.

## Discussion

This report describes the short- and long-term outcome of nine cats with IMPN treated with hIVIg. Cats who were chronically affected or had acute and severe neurological signs were treated with hIVIg. Eight cats showed rapid clinical improvement after administration of hIVIg and were ambulatory for more than five steps within a median of 4 days. All cats recovered with only minor signs of weakness after a median of 14 days (partial recovery) and completely after a median of 20 days. Four cats experienced relapses, but all nine cats ultimately recovered.

IMPN is a frequent cause for generalised LMN signs in cats. The clinical course and histopathological findings are consistent with immune-mediated inflammatory aetiology with parallels to human immune-mediated neuropathies (i.e. GBS, CIDP, nodo- and paranodopathies).^1–3,16,17,25^ Most recently, acute motor axonal neuropathy, a subtype of GBS, has been proposed as the most likely mechanism of IMPN in cats.^2^ Multiple episodes of weakness occur in 28–35% of those affected.^1,2^ In some cases, cats may not recover, die or be euthanased because of IMPN.^1,26^

Human IVIg resulted in improvement and return to ambulation in 8/9 cats with IMPN who were acute and severe or chronically affected (Figure 2). One cat failed to respond. The responder cats were able to walk more than five steps without support in a median of 4 days. In the present cohort, the median times from presentation to partial or complete recovery were 14 and 20 days, respectively, which is broadly comparable to previously reported median recovery times of 4–6 weeks in cats with IMPN.^1,2^ Chronically affected cats treated with hIVIg reached complete recovery after a median of 18 days, although the small sample size precluded meaningful statistical evaluation. Previous studies, including those involving cats receiving other treatments or no treatment, have also reported rapid improvement in some cases, indicating that spontaneous or treatment-associated recovery may occur.^1,2^ Therefore, although the rapid improvement observed in several cats, particularly chronically affected cats, is encouraging, comparisons with previous studies remain indirect and should be interpreted cautiously because of differences in baseline severity, outcome definitions and cohort size. The relapse rate in this cohort was similar to that reported in previous studies.^1,2^ Remission without recurrence of signs after hIVIg was seen in four cats. The four other cats (two acutely; two chronically affected) relapsed 10–43 days after hIVIg treatment, but remained ambulatory, with less severe clinical signs compared with the initial episode.^1,2^

In humans, treatment with hIVIg is an established and widely applied treatment modality in autoimmune neuropathies.^9,16,17,27^ Human IVIg exerts complex immunomodulatory and anti-inflammatory effects through multiple mechanisms, including fragment crystallisable-gamma (Fcγ) receptor blockade, contributing to the rapid control of autoimmune and inflammatory processes, and induction of inhibitory FcγRIIB signalling, accelerated clearance of pathogenic antibodies, modulation of cytokine networks, interference with complement activation and neutralisation of autoantibodies.^28^ In addition, hIVIg influences B- and T-cell activity, promotes regulatory T-cell activity and modulates dendritic cell function.^1,2,28^ These mechanisms may be relevant in feline IMPN, as anti-ganglioside antibodies, including anti-GM2 and anti-GalNAc-GD1a IgG antibodies, are present in affected cats, supporting a potential role for humoral immune mechanisms.^3^

In antibody-mediated neuropathies such as GBS, hIVIg is thought to neutralise pathogenic antiganglioside antibodies through idiotypic/anti-idiotypic interactions, inhibit antibody-mediated cytotoxicity, and modulate B-cell activity and antibody production. Human IVIg contains pooled IgG with diverse idiotypic and anti-idiotypic specificities, which may neutralise pathogenic autoantibodies and modulate B-cell activity. Therefore, if anti-ganglioside antibodies contribute to peripheral nerve injury in feline IMPN, their modulation by hIVIg could partly explain the clinical improvement observed in the present cohort. Infused IVIg also has been shown to reach peripheral tissues, including muscle fibres, end-plate regions, distal axonal terminals and nerve roots lacking a blood–nerve barrier.^29^

Human GBS presents acutely and reaches its clinical nadir within 2 weeks and not later than 4 weeks.^27^ Conversely, CIDP is defined by a progressive phase exceeding 2 months and a chronic relapsing course; acute-onset presentations can initially mirror GBS.^27^

Treatment guidelines for immune-mediated peripheral neuropathies in human medicine provided the rationale for trialling hIVIg in cats with suspected IMPN. The European Academy of Neurology/Peripheral Nerve Society recommend hIVIg or plasma exchange as initial treatment options for GBS in non-ambulatory patients, and hIVIg for CIDP with pure motor signs.^16,17^ Treatment with corticosteroids is not recommended for GBS.^16,17^ In CIDP, hIVIg and corticosteroids are both considered first-line treatment options.^16^ Both GBS and CIDP may follow chronic or relapsing courses and treatment responses in humans are variable; improvement is not invariably achieved. The standard hIVIg protocol of CIDP^16^ or treatment-related fluctuations in GBS^17^ involves repeated hIVIg administration every 4 weeks, with earlier retreatment in patients who deteriorate clinically.^9^ Thus, these recommendations served as the catalyst for using hIVIg as a treatment in the present feline cohort, although they do not provide direct evidence of efficacy in cats. In a previous observational study, treatment with hIVIg showed promising results in dogs with ACP. Of 16 dogs with ACP, 10 walked independently within 6–13 days (median 8) after treatment with hIVIg and were considered responders.^13^

Four cats initially improved after hIVIg but then relapsed. This raises the question of whether some cats could benefit from multiple courses of hIVIg. One cat (cat 5) received a repeat hIVIg course and improved again to grade 6 approximately 3 weeks after the second hIVIg course, which was slightly longer compared with the initial treatment. The spontaneous improvement of cat 1 after relapse is clinically particularly relevant, as this had been the most severely affected cat at initial presentation, with tetraplegia. This observation suggests that responses to interventions in cats with IMPN need careful interpretation. Even episodes in cats with initially severe disease may improve without treatment. Notably, some cats in this cohort appeared to respond to prednisolone during relapse (cats 4 and 6). The cat (cat 9) who initially failed to respond to hIVIg also improved with prednisolone. This cat was 11 years of age, had evidence of significant inflammation of intramuscular nerves on muscle biopsy, was responsive to treatment with prednisolone and had significant electromyography changes (indicative of denervation). This case (cat 9) certainly falls under the same umbrella as IMPN but may be different. Previous larger studies also noted that a small proportion of affected cats with IMPN (10%) may be aged >5 years.^1^ These cats may represent a different subgroup of IMPN. Other authors have anecdotally described clinical improvement in individual cats with IMPN treated with corticosteroids.^2,30,31^ Future studies comparing corticosteroids and hIVIg are warranted, and a trial with corticosteroids might be indicated before using hIVIg. It is interesting that corticosteroids are used as first-line treatment in CIDP but not in GBS patients.^16,17^ A controlled trial comparing corticosteroids, hIVIg and supportive care alone in cats with IMPN should be considered.

The lack of a control group is a major limitation of the present study. To provide context, the outcome is compared descriptively with previous feline IMPN cohorts.^1,2^ Other limitations of this study are the small cohort size and the retrospective nature. The small cohort size contributes to variability and limits statistical comparison to data from other researchers. Furthermore, electrodiagnostics were not consistently performed. Electrodiagnostic results may help to characterise abnormalities (e.g. denervation, nerve conduction) but are not specific for a particular cause. In practice, clinical signs and history, together with the exclusion of toxicities, metabolic causes and some myopathies/neuromuscular transmission disorders, form the basis of diagnosis in the majority of cases of IMPN, and electrodiagnostics is not necessarily required.

The major drawbacks of hIVIg treatment are the substantial costs, availability, costs of hospitalisation and lack of proven prevention of relapse. The cats with IMPN in this cohort were hospitalised and closely monitored during and after the hIVIg CRI, and none developed treatment-related complications. In previous reports, cats tolerated hIVIg well, and no major adverse effects were observed.^19,21,22^ Nevertheless, rare but serious adverse effects have been reported in humans, primarily thrombotic events. Additional adverse effects in humans included renal impairment, arrhythmias, aseptic meningitis, haemolytic anaemia and transfusion-related acute lung injury on rare occasions.^32^ Therefore, the potential for serious adverse effects should be considered, and a careful risk–benefit assessment is warranted. Post-treatment laboratory monitoring is recommended to ensure that haematological abnormalities are not overlooked, given that haemolysis occurred after hIVIg treatment in a dog.^33^

## Conclusions

Human IVIg was well tolerated in this small number of cases and rapid clinical improvement was seen in the majority of treated cats. Based on this study, it may be considered as a useful treatment in selected cases of cats with IMPN. It may benefit cats who are chronically affected, but stronger evidence is needed to promote its use, particularly early in the clinical course. A larger prospective study, along with a comparison with no treatment or corticosteroids, is needed to further assess its effectiveness.

## Supplemental Material

**Figure:** Video 1: Chronically affected cat (cat 6) with immune-mediated polyneuropathy at initial presentation (grade 2)

**Figure:** Video 2: Neurological examination (cat 6) demonstrating reduced to absent withdrawal and patellar reflexes

**Figure:** Video 3: The same cat (cat 6) 4 days after initiation of treatment with human intravenous immunoglobulins, showing marked improvement in ambulation (grade 4)
